# Cell injury, retrodifferentiation and the cancer treatment paradox

**DOI:** 10.1007/s13277-015-3981-2

**Published:** 2015-09-07

**Authors:** José Uriel

**Affiliations:** 1 Visiting scientist: Apoptosis, Immunity and Cancer Group, Department of Biochemistry and Molecular and Cell Biology, University of Zaragoza, Zaragoza, Spain; 2 50 rue Corvisart, 75013 Paris, France

**Keywords:** Cell injury, Retrodifferentiation, Foetal characteristics of cancer, Tumour heterogeneity, Steam cells and cancer stem cells, Genomic analysis, Cell killing therapies

## Abstract

This “opinion article” is an attempt to take an overview of some significant changes that have happened in our understanding of cancer status during the last half century and its evolution under the progressive influence of molecular biology. As an active worker in cancer research and developmental biology during most of this period, I would like to comment briefly on these changes and to give my critical appreciation of their outcome as it affects our knowledge of cancer development as well as the current treatment of the disease. A recall of my own contribution to the subject is also included. Two subjects are particularly developed: cell injury and cell-killing therapies. Cell injury, whatever its origin, has acquired the status of a pivotal event for the initiation of cancer emergence. It is postulated that cell injury, a potential case of cellular death, may also be the origin of a process of stepwise cell reversion (retrodifferentiation or retroprogrammation) leading, by division, mature or stem cells to progressive immaturity. The genetic instability and mutational changes that accompanies this process of cell injury and rejuvenation put normal cells in a status favourable to neoplastic transformation or may evolve cancer cells toward clones with higher malignant potentiality. Thus, cell injury suggests lifestyle as the major upstream initiator of cancer development although this not exclude randomness as an unavoidable contributor to the disease. Cell-killing agents (mainly cytotoxic drugs and radiotherapy) are currently used to treat cancer. At the same time, it is agreed that agents with high cell injury potential (ultraviolet light, ionising radiations, tobacco, environmental pollutants, etc.) contribute to the emergence of malignant tumours. This represents a real paradox. In spite of the progress accomplished in cancer survival, one is tempted to suggest that we have very few chances of really cure cancer as long as we continue to treat malignancies with cell-killing therapies. Indeed, the absence of alternatives to such treatments justifies the pursuit of current procedures of cancer care. But, this should be, precisely, an urgent stimulus to explore other therapeutic approaches. Tumour reversion, immunotherapy, stem cell management and genomic analysis of embryo-foetal development could be, among others, appropriated candidates for future active research.

## Biology beyond the gene

Fifty years of brilliant success, from the discovery of the helical structure of DNA to the sequencing of the human genome, has made molecular biology an irreplaceable paradigm of biological sciences. Nevertheless, the molecular analysis of living processes has shown its limitations. In the course of an open talk about “biology beyond the gene” (Le Monde, 25 September 2004) between François Jacob, Nobel prize winner in Physiology and Medicine (1965) and Pierre Sonigo, Laboratory Director at the Institut Cochin (Paris), an agreement was gained on the statement that modern Biology has failed “to elucidate the true essence of living matter” and also failed “to work out the therapeutic tools able to solve the challenges opened up by modern Medicine” (see also “The Myth of Biotechnology Revolution” [1]).

Eleven years later, the same analysis remains valid. We do not know how to cure cancer nor the great majority of degenerative diseases such as diabetes, obesity, cystic fibrosis, Alzheimer’s disease, Parkinson’s disease and many others. However, things have improved and we know how to treat them with varying success. The terms “successful treatment” and “cure” are not equivalent. During the first half of the twentieth century, the mortality due to tuberculosis was still high although many patients were saved from dying thanks to the therapies available at that time. The disease begun to be really “cured” with the arrival of antibiotics that enabled the possibility to clean specifically the infecting bacteria.

Molecular biology continues to be indispensable, but insufficient to overcome numerous biological and medical problems.

## Cancer, causes or consequences

After a century of thinking and research about cancer, the old clinical idea that “cancer is a potentially malignant tumour” remains valid and provides not much less information than many sophisticated definitions. During the past half century, our learning of the molecular, cellular and microenvironmental patterns involved in the biology of cancer has increased almost exponentially and represents today an impressive corpus of knowledge.

Different cell abnormalities, functional and structural, genetic and/or epigenetic, have been associated with cancer emergence and progression, but none of them can give a pertinent explanation of the events, simultaneously “necessary” and “sufficient”, to provoke the neoplastic transformation of cells. On the other hand, we ignore the timing of cellular changes (i.e., somatic mutations) or the sequence of events (i.e., the evolving of cancer stem cells) giving rise to the critical malignant phenotype. It is not surprising, since we are concerned with a disease that can be lethal in a few months or after one or more decades, or even never, that survivors may die from a different pathology, unrelated to cancer. This testifies to the enormous complexity of human malignancies.

For more than a century, many causes and factors have been proposed to account for cancer, often related with the branch of biomedical research prevailing at the time that those concepts were postulated. Successively or simultaneously, cancer has been attributed to problems with metabolism, the dysfunction of some enzymes, weakness of the anti-tumour activity of the immune system, a disordered process of cell differentiation, an infection with oncogenic viruses, an aberrant programming of normal genes, the activation of tumour genes, the expression of genes involved in the positive (proto-oncogenes) or negative (suppressor-genes) regulation of cell growth, the unbalance of anti-apoptotic and pro-apoptotic pathways in favour of the former, the failure of the homeostatic control of tissue-specific stem cells, and, more recently, to genomic instability at the origin of genomic aberrations. The list is, indeed, incomplete but each one of these proposals bears part of truth. The main difficulty in making a choice among the suggested concepts of the origin of cancer lies in how to discriminate causes that are upstream of the oncogenic transformation from those which are in fact the consequence of the cancer.

Recently, coincidental with the “genomic era”, the following assertion was advanced [2]: “all cancers are caused by somatic mutations; however, understanding of the biological processes generating these mutations is limited”. The genomic instability that underlines the high rate of mutations found in many tumours supports this statement, but its limitation probably results from the fact that cancer is firstly a disease and as it stands should be explored at different levels of complexity. I would like, in the present paper, to reflect on some major factors that, during the last 60 years, have become accountable for cancer emergence and development, including particularly, stem cell differentiation/retrodifferentiation pathways that, in my opinion, seem to play a non-negligible part in the initiation, progression or regression of malignancies.

## Embryo-foetal characteristics of cancer

This subject, the resemblance between neoplastic and embryonic tissues, that attracted the attention of biologists for more than a century, has lost part of its previous interest. However, it is worth remembering some facts, particularly because recent experience has revived interest in the foetal patterns of gene expression in cancer as well as on the activation of foetal genes in animal and human malignancies.

A series of acquired biological capabilities characterising the stepwise development of malignant tumours has been proposed [3, 4] as “an organising principle for rationalising the complexities of neoplastic disease”. They include sustaining proliferative signalling, evading growth suppressors, resisting cell death, enabling replicative immortality, inducing angiogenesis and activating invasion and metastasis. These hallmarks of cancer constitute an excellent compendium of the molecular basis that sustains our present knowledge of the biology of malignant tumours. Interestingly, most of them have their counterparts in embryo-foetal development. Thus,

1. Embryos and neoplasms first appear as cellular populations with “self-sufficiency in growth signals” and a real autonomy compared to the cellular dynamics and organisation of the hosts.
2. Cell death regulation is essential for numerous life processes. In fact, the mechanisms that control the different types of cell death, apoptosis being the best studied, remain to be well delineated. Nevertheless, it is obvious that in malignant as well as in embryo-foetal tissues, the balance between apoptotic and anti-apoptotic signals must be in favour of the second in order to achieve their common destiny, an increase in cellular mass.
3. Both ontogenic and neoplastic development progress thanks to a high proliferative potential that mimics cell immortality. Cancerous cells maintained in culture show similar behaviour. This contrast with the short number of divisions proper to normal adult cells before they enter senescence and the dying processes. It is true that telomerase, the enzyme that adds telomere repeats onto chromosomes, plays an important role in the acquisition of cell immortality. Telomerase is functional in the great majority of malignant neoplasms but has also been demonstrated to be fully active in all human foetal tissues examined and to decline abruptly after birth [5].
4. To induce angiogenesis, it is a requisite for all growing cell populations in order to supply the requirements of nutrients and oxygen as soon as a nascent cell mass has attained a critical size. This also occurs in developing tumours and embryos.
5. The ability to invade nearby structures and to migrate far from their place of origin is a property shared by both malignant and embryonic cells. A remarkable case is provided by skin melanomas, the cells of which can spread to distant organs. Similarly, normal melanoblasts, cells derived from the neural crest, a primordial embryonic structure, colonise the skin and hair follicles of mammals during the gestational period to later become differentiated melanocytes and the potential source of oncogenic transformation. When this occurs, melanoma cells utilise regulatory signals and pathways characteristics of embryo-foetal development and regeneration [6].

An emerging hallmark, recently proposed [4], concerns the classical observation of Warburg [7] that most tumours show low respiration rates and, when metabolising glucose, produce lactate at higher rates than do normal adult tissues. This led him to conclude that damage to the respiratory process of malignant growing cells and the adoption of a fermentative pathway to derive energy necessary for survival were the cause and the mechanism of cancer. Only much later, Villee [8] did demonstrate significantly high aerobic glycolysis in all human embryonic tissues of the gestational period studied. That is, the pathway to derive energy metabolism is a hallmark common to cancerous and embryo-foetal cells.

A different approach to the question of the relationship between cancer and developmental biology was taken in 1945. Greenstein concluded from his studies that “tumours tend to converge to common enzymatic patterns” and that in certain cases, these patterns resemble those of foetal tissues [9]. The question was brought up again in 1963 after the discovery by electrophoretic and or immunodiffusion techniques of an aldolase A of foetal type in soluble extracts of rat hepatomas [10] and of a protein (alpha-fetoprotein) in the sera of adult mice bearing chemically induced hepatomas [11]. The finding was confirmed in the case of human hepatocarcinomas [12]. Soon later, the presence of another foetal protein, called carcino-embryonic antigen, was demonstrated in the serum of patients with colonic cancer [13, 14].

The possibility of revealing the presence of a tumour by the single analysis of a serum sample opened a still active domain of medical research and clinical practice in oncology, that of “tumour markers.” It also focused the interest of oncologists on embryo-foetal development and its role in the emergence of malignant tumours (see review by Uriel [15]).

Unlike to the intensive work undertaken for decades to elucidate the molecular basis of cancer, its comparative search in embryo-foetal development was seldom undertaken. Recent work from Spike et al. [16] has shown that a mammary stem cell population, identified and characterised in late embryogenesis, exhibits gene expression profiles with significant similarities to basal-like breast cancer subtypes. Also, Ben-Porath et al. [17] have found that histologically poorly differentiated human tumours of various types show preferential overexpression of genes normally enriched in embryonic stem cells. In breast tumours, this embryonic cell-like signature was associated with high-grade oestrogen-receptor-negative tumours [17]. Again, a molecular switch that appears to turn on some genes known as promoters of rapid growth in foetal and early postnatal life is also reactivated in cancer cells [18].

Taken together, these observations confirm the importance of the foetal characteristics of cancer as a source of useful information that can contribute to better understanding of the biology of cancer at molecular, cellular and micro-environmental levels. For instance, it would be very interesting to know the genomic profile of cells during embryo-foetal development. It would not be surprising to find significant analogies in the expression of genes proper to malignant elements.

## Differentiation/retrodifferentiation pathways

The interest in cancer research for cell differentiation was a consequence of the demonstration of the embryo-foetal properties associated with malignancies of different origin. Thus, late in the 1960s, Pierce [19, 20] concluded that the target cell of the majority of cancers was a pluripotent embryonic cell (stem cell) and Potter [21, 22] considered that neoplasms were the result of the interrupted differentiation of a population of embryonic stem cells. A different, although not exclusive, concept of oncogenic transformation was proposed by Uriel [23, 24]. Such transformation can occur during the process of cell rejuvenation (retrodifferentiation) that may affect normal elements of many tissues following cell injuries of varied nature. Uriel introduced the term of “retrodifferentiation” [23]^1^, instead of that of “dedifferentiation”, in order to emphasise the “stepwise and ordered character” of the process. The reversion of adult elements towards foetal-embryonic states is thus considered as a broadly inverted sequence relative to that of differentiation.

The impact of harming factors in the nucleo-cytoplastic structures of the aggressed cells may have, at least, three different consequences: (a) the damage has been very faint, and the natural resources of cell repair can restore the initial status of the cell; (b) the injury has overcome the cell capability of life maintenance, and the cell dies through necrotic or apoptotic processes; and (c) the cell disorder has been moderate, and a third option is opened up—cell retrodifferentiation. The latter appears then as an alternative to the cell’s risk of suffering irreversible deleterious changes. It enables cells, adult or stem cells, to revert temporally towards younger stationary states better adapted to stressing environmental conditions. The change is advantageous because when cells differentiate, they expend energy and bind information content to build new structures and to develop new functions. On the contrary, cells undergoing retrodifferentiation lose information content since they self-simplify their structures and thereby evolve energy that can be used to increase their growth potential. In other words, differentiation is a negentropic process while retrodifferentiation is an entropic one [24]. The latter is consequently a thermodynamically favourable and less costly change. The question is not merely academic, cells leaving a stationary state and facing the alternative between the two bi-directional pathways (differentiation vs retrodifferentiation) will be, in the absence of differentiation impulses, naturally inclined to revert through more immature states. This may explain why neoplasms evolve, early or late, to phenotypes of higher growth potential and malignancy. On the other hand, the rejuvenation process of injured adult or stem cells contributes to increase the number of dividing cells of a tissue and thus the risk of cancer emergence as a consequence of lifestyle factors, including those derived from an aggressive cancer treatment (see also “The future of cancer care”). According to this, the part played by the randomness versus the lifestyle (2/3 to 1/3) in cancer [26] may change or even be reversed by the extra-cell divisions induced after cell injury. One property often associated with immature cells, either normal or cancerous, undergoing retrodifferentiation is their ability to bind and to internalise alpha-fetoprotein (AFP) via the expression of specific receptors [27]. Another example of how normal and neoplastic non-stem cells may evolve further in the way of retrodifferentiation is provided by the work of Chaffer et al. Another example of how normal and neoplastic non-stem cells may evolve further in the way of retrodifferentiation is provided by the work of Chaffer et al. [28]. Studying cultures of normal and neoplastic mammary epithelial cells (HMECs), they could demonstrate that these differentiated elements can spontaneously revert to a stem cell-like state.

The preferential evolution of cells, normal or cancerous, towards retrodifferentiated states when subjected to damaging or stressing agents was, to my knowledge, never considered, neither in the elaboration of the current concept dealing with the role of stem cells in oncogenesis, nor in the pivotal problems associated with cancer heterogeneity and regenerative medicine. For the latter, it may be a real difficulty able to disturb, to slow down or to arrest the terminal differentiating steps necessary for successful tissue repair or organ regeneration (see also below under “normal and cancer stem cells”)

## Normal and cancer stem cells

Studies on stem cells (SCs) have received considerable attention in the last decade related with both cancer development and regenerative medicine. Stem cells are currently characterised by their ability to self-renew indefinitely and to differentiate or, as commented above, to retrodifferentiate into a variety of specialised cell types. The terminology used to describe SCs is slightly complex due to the fact that it has been established, either on a conceptual basis (totipotent “embryonic stem cells”; unipotent, pluripotent or multipotent “adult stem cells”), or on an operational basis such as those referring to the tissue from whence they arise (mesenchymal-, skin-, intestinal-, hair follicle-stem cells, etc.). A great impulse for stem cell studies was obtained by the discovery of the method to produce pluripotent stem cells (iPSCs) from murine adult somatic cells [25]. This was initially obtained by the introduction of four specific genes and later by other genetic manipulation of cells of different origin, including human. These procedures that avoid the controversial use of human embryonic stem cells for experimental purposes have considerably facilitated studies on regenerative medicine.

Numerous different phenotypes of normal stem cells have been characterised and isolated in a variety of adult tissues from invertebrates to humans. These “tissue-specific stem cells” are frequently grouped forming sort of “niches” in the vicinity of, and in close interaction with, a microenvironment of normal adult resting cells. Normal stem cells play an important role in physiological processes accompanying the renewal, maintenance or repair of tissues and cellular structures. One important point to be elucidated is why tissue-specific stem cells, provided with an assumed high growth potential, remain in a stationary state. We ignore the question of what are the signals or the microenvironmental conditions that drive these inert stem cells towards more differentiated or retrodifferentiated states.

More recently, a new interest in the stem cell area of research has come from the so-called cancer stem cell (CSC) hypothesis. This hypothesis is based on the finding within tumours of malignant cells with the characteristics of adult stem cells and suggests that they derive from the neoplastic transformation of tissue-specific normal stem cells from different origin and probably represent the true drivers of cancer development, particularly of solid tumours (see reviews by Nguyen et al. And Iqbal et al. [29, 30]). Cancer stem cells have been characterised and isolated from human malignancies. Most of the information supporting the CSC hypothesis has been obtained; however, from experiments done after transplantation of isolated CSCs into immune-suppressed or immune-deficient mice. As discussed below, the simple fact of isolating CSCs from tumour biopsies already implies a harmful process. There is a great probability that cells having escaped alive from such treatments have already experienced damage able to induce differentiation/retrodifferentiation events and/or to facilitate genomic or other nucleocytoplastic changes that could lead sooner or later to the emergence of new malignant clones not present in the original tumour. Also, the growth of human CSCs in immune-depressed mice may contribute, via selection pressure of different homeostatic conditions, to unnatural erratic developments. Analogous restrictions may be addressed to assays performed using long-time established lines of mouse or human tumours. The conclusions derived from such experiments cannot be accepted without caution. In fact, recent work in this area has shown that the CSC concept is more complex than initially considered. Thus, examples have been found in the existence of abnormal clones of cells with mutations of known oncogenic potential that, nevertheless, does not manifest malignancy for years. It has been suggested applying to them the term “neoplastic stem cells” and to restrict the term “cancer stem cells” to those generating fully malignant clones [31].

The CSC concept, although controversial [32], is of great interest because it brings into focus the pivotal role of cell differentiation in neoplastic transformation and/or in the further evolution of transformed cells giving rise to the emergence of new clones of cells whose eradication could be determinant for a successful cancer treatment [33]. In spite of the criticisms that can be made of this concept, stem cells and cell differentiation have become and should continue to be major topics in cancer research.

## Cancer heterogeneity

**Fig. 1:**
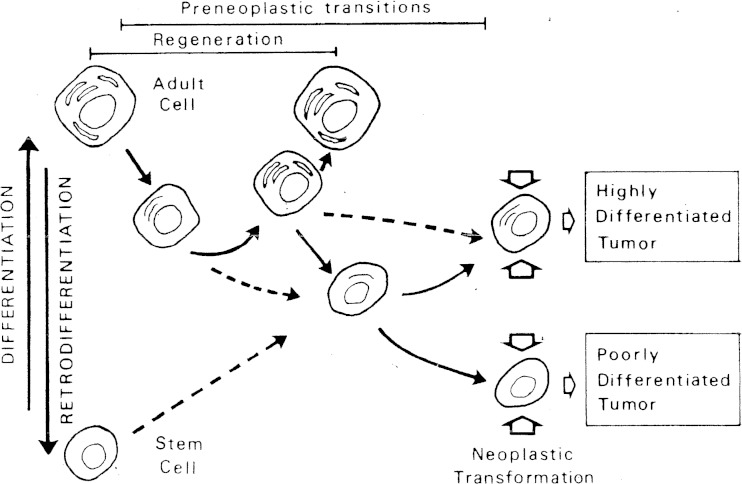
Schematic representation of hypothetical neoplastic development arising in adult tissues. A mature liver parenchymal cell enters retrodifferentiation under hepatic injury. The initial transition follows distinct pathways: either it can be “counter balanced” by a process of re-ontogeny which restores the phenotypic properties of the mature cell (regeneration) or it can persist “unbalanced” and even progress, the cell travelling through various stages of “rejuvenation” until the neoplastic transformation takes place. *Dashed arrows* indicate alternative routes of stem cells that emphasise the plasticity of the hypothetic model. Several phenotypes of malignant clones may coexist in the same tumour (reproduced from Uriel [15])

The heterogeneity of tumours was already well known and characterised by morphologic criteria thanks to the careful work of histopathologists throughout the twentieth century. In 1976, Nowell proposed, for the first time, that progression of tumours might result from acquired genetic instability within the original clone and the accumulation of genomic alterations that could favour the growth of cloned variants with survival advantage [34]. The possibility of intra-tumour heterogeneity was also anticipated on the basis of differences encountered between rat liver regeneration and oncogenesis [15] (see also Fig. 1). The presence of multiple clones derived from a single tumour was attributed, at least in part, to the level of the differentiation step where the neoplastic transformation took place or remained stationary.

After the sequencing of the human genome in 2001, there has been interest in genomic analysis of tumours with the idea of characterising somatic mutations that occurred during cancer emergence and progression and then developing drugs or procedures better adapted to the treatment of a given tumour as well as discovering new biomarkers with higher discriminating ability (for reviews, see [35, 36]). Unfortunately, the recent demonstration of the heterogeneity of the genomic profile in different areas of a single malignancy and between the original tumour and its metastasis has tempered the hope of rapid progress in personalised treatments. The same limitations concern the development of treatments based on biomarker’s data obtained from a single biopsy [37]. Genomic profiles and biomarkers can also change with the evolution in time of the clones derived from the original tumour, due in part to the selection pressure resulting from the use of different treatments.

Nevertheless, the awareness of intra- and inter-tumour heterogeneity is rapidly having a considerable impact in current cancer research because it represents a major contribution to the biology of cancer and in medical practice due to its consequential effects on cancer management (see reviews by Russnes et al. and Sonner et al., 2012 [36]. The multiplicity of samples that need to be analysed at one time from a single patient and at several times during the evolution of the patient’s tumour makes the development of adequate drugs, or the choice of other relevant treatments, an enormous and, at present, almost insurmountable task [38]. Moreover, the already elevated costs associated with cancer therapies will be further increased by the eventual use of such procedures.

## Regeneration versus neoplastic transformation

The irreversibility of the adult cell state has in the distant past been a firmly held opinion by many embryologists. Today, as experimental evidence has accumulated, there is no formal argument against the assumption that embryonic reversion is potentiality inherent to all somatic cells of an organism as long as their genetic information content is preserved. The ability to revert may, however, vary among cells of different organisms or from one cell species to another in the same individual [39, 40]. On the other hand, varied observations emphasise the central role of retrodifferentiation in regeneration, wound healing and neoplastic growth (Fig. 1) [24, 41].

The link between regeneration and cancer was postulated in 1935 [42], and the topic periodically revisited since then. In mammals, both processes can start as a response to cell or tissue injuries of external (e.g. physical harm, cytotoxic agents, viruses) or internal (hormones, ageing) origin. Both are characterised by the presence of immature growing cells: tissue-specific stem cells in regeneration or tissue repair and cancer stem cells in neoplasms. Both processes progress, among other events, by the gene expression of foetal type proteins and enzymes. The origin of these stem cells remains, nevertheless, uncertain. The question has been raised whether they preexist in significant quantities as normal tissue components (stem cell niches) or are induced by cell injury and neoplastic transformation. On the other hand, the presence of stem cells in regenerating and neoplastic tissues could be also inferred from the hypothesis [24] that cell injury triggers retrodifferentiation and consequently a process of cell rejuvenation with the appearance of stem cells of a different degree of immaturity.

This alternative has recently found experimental confirmation in vitro and in vivo. Thus, Chaffer et al. [28] have identified “a subpopulation of basal-like human mammary epithelial cells that spontaneously dedifferentiate into stem-like cells”. Moreover, neoplastic transformation enhances spontaneous conversion, so that adult, non-stem cancer cells give rise to cancer stem cells in vitro and in vivo. Also, the work of Abad and co-workers [43] has produced evidence that teratomas from multiple organs emerge in mice after transitory induction of four transcription factors concomitant with the presence of circulating iPSCs showing characteristics very close to those of embryonic stem (ES) cells. This experiment demonstrates again the possibility of in vivo retroprogramming of adult cells.

The relationship between tissue repair, regeneration and cancer development represents a serious challenge for future progress in regenerative medicine because, as discussed above, terminal steps of cell differentiation necessitate the expenditure of energy and bound information content to acquire their final structures and functions characteristic of operational adult tissues and organs. This process competes with the expected increase of rejuvenation potential of cells approaching the adult state.

## The cancer treatment paradox and the strategy of killing cancer cells

The comparative analysis of past and current anti-cancer treatments and the accumulated knowledge after more than a century of research and medical practice in cancer leads to a paradoxical situation. There is agreement on the major external causes leading to the emergence of human malignancies: ionising radiations, ultraviolet light, environmental pollutants released by vehicles and industrial processes, pesticides, tobacco or alcohol abuse, etc. Beyond a given threshold of concentration or of cumulative effect, they become potentially oncogenic due to their common ability to damage cells by contact, inhalation, ingestion or other ways. Regenerative cycles, involving tissue-specific stem cells, in response to cell wear or ageing, occur regularly in many tissues, such as skin, respiratory and digestive tracts and can be the target of oncogenic transformation. Cell injury and cell damage, either from external or internal sources, are concomitant with all these events and seem to be a requisite for cancer initiation. Therefore, the current treatments (surgical eradication, radio-, chemo-, immune- and hormone-therapies) supposed or intended to “cure” cancer have, as their main and common purpose, the killing or the removal of the largest number of cancer cells. In other words, the agents that may initiate malignant tumours and those intended to treat them are both essentially cell injury agents. That is the paradox.

If the reasoning outlined above (“Differentiation/retrodifferentiation pathways”, “Normal and cancer stem cells” and “The cancer treatment paradox and the strategy of killing cancer cells” sections) is correct, cell injury can entail differentiation/retrodifferentiation processes that preserve the survival of some of the aggressed cells, but puts them in a state of genetic instability, then cancer treatments that damage cells may become double-edged weapons. On one hand, they destroy malignant cells while on the other hand, they can injure and, consequently, induce divisions and mutational changes in cells that have escaped previous treatments. This may involve normal and cancer stem cells, dormant micrometastasis or, even, healthy active and resting adult cells of the same or different tissues. In all cases, there is the risk of the genesis, via differentiation/retrodifferentiation stimulus, of new malignant clones with different sensitivity to the previously used anti-cancer agents. Tumours of different histological origin, particularly of the hemo-poietic system, may also emerge for the same reason. The latter have been observed and found statistical confirmation, in cases of acute myeloid leukaemia (AML) following breast cancer treatment with chemical drugs. Contrary to the preceding findings [44], recent studies have pointed out the increased risk for both AML and myelodysplastic syndrome post-radiation treatment [45]. The benefits awaited and often really gained with the use of cytotoxic agents in cancer care may, thus, become counter-productive.

The great intra- and inter-heterogeneity, both spatial and temporal (for a review, see Russner et al. [36]) of human tumours, in long-term cancer care and the use of successive treatments, most of them cytotoxic, are becoming lethal for a high proportion of malignant elements. At the same time, there is increasing probability of cancer progression that can lead to more aggressive clones of tumour cells and finally to the terminal phase of the disease.

In face of this situation, one is tempted to suggest that we have very little chance, if any, of really “curing” the great majority of cancers, particularly those of solid tumours, as long as the main, if not the sole, treatment strategy continues to be that of killing cancerous cells.

## The future of cancer care

Should we infer from these considerations that the present therapies for fighting cancer should be stopped? Certainly not, because of the real progress in the choice and use of drugs and radiation to target tumours and, above all, because many comparative studies have proven that current approaches efficiently prolong the survival of patients with a large variety of tumours or even to make cancer a chronic disease. Also, we know better than in the past how to soften and/or to prevent the collateral effects of current treatments although heart damage from cancer chemotherapy and/or radiation continue to be a serious problem in clinical practice [46]. On the other hand, we have not found anything to replace such therapies. In the history of medicine, a paradigmatic therapy was never abandoned before another one had proven its unquestionable advantage. This is the challenge for future cancer care. In any case, this should not be an impediment to looking insistently for different approaches, and among them are the following.

### Tumour reversion

Is neoplastic transformation an unchangeable event? The possibility to reverse the cancer status has fascinated oncologists for more than a century. Evidence of phenomenon in plants was first supported by the pioneer work of Armin Brawn [47]. Later on, in his excellent review devoted to this topic [48], he extended the analysis of tumour regression to several animal species, man included. Two typical examples are the induced differentiation of mouse teratocarcinoma into multiple mature tissues and the spontaneous regression of childhood neuroblastomas. An experiment providing evidence that the gene information present in a cancer nucleus may be not irreversible was performed by McKinell et al. [49].

Adenocarcinomas were induced in a triploid line of frogs. Nuclei from the tumour cells were injected into enucleated normal frog eggs. A significant number of triploid swimming apparently normal larvae or tadpoles were produced. It appears from these studies that despite serious genomic imbalance, malignancies may be completely reversible and led Armin Brawn to conclude that “there is certainly no longer any good reason for believing that nuclear gene function in a cancer cell is beyond hope of correction…” [48].

Subsequently, studies at the microenvironmental and molecular levels have confirmed the possibility of the experimental or therapeutic reversion of the malignant status. Several laboratories interested in the development of normal and neoplastic cells used matrigel tridimensional cultures of cells isolated from breast and other human cancers to analyse tumour reversion. They arrived at the conclusion that “the correction of 1 or 2 signalling defects can revert tumour cells to a normal phenotype, both in vivo and in vitro” (see review by Kenny and Bissell [50]).

In humans, the reversion of malignancies of the hemopoietic system has yielded the best clinical benefits. Thus, treatment of acute promyelocytic leukemias with retinoid acid alone or in combination with chemotherapy may result in complete induction of differentiation of promyelocytes to mature cells [51]. Relapses after a short period of remission were, however, frequently noticed.

A different approach was undertaken by the group of Amson and Telerman. The purpose of their research was to understand the molecular program of tumour reversion and its clinical application. They first isolated revertant cells from breast and other tumours followed by the differential analysis of gene expression between the parental cancer cells and the derived revertants [52]. At least 300 genes were implicated in the reversion process. Among them, it appeared that translationally controlled tumour protein (TCTP) is a key gene that needs to be switched off before a malignant cell proceeds to revert [53, 54].

The rarity of spontaneous or induced regression of human cancers and the small number of laboratories currently involved in these studies should not be a reason to neglect this approach. On the contrary, coordinated research work in this area at molecular, cellular and micro environmental levels should be encouraged for the benefit of both the insight into our knowledge of the biology of cancer and the possible eventual successful treatment of the disease.

### Cell differentiation management

This is a corollary of the preceding comments. As discussed above, normal stem cells moving along differentiation//retrodifferentiation pathways are currently derived under strict homeostatic conditions in renewing, expanding and regenerating tissues (skin, hemopoietic system, liver, etc.). Both pathways are also involved in cancer development and progression and could represent the basis for an alternative cancer treatment different from that of cell-killing therapies currently used to fight malignant tumours [52]. Unfortunately, to date, we do not have enough information on the molecular events, the forces involved and the ways to manage cell differentiation and its alternative way, retrodifferentiation or retroprogrammation. Research work in this area, which can be extended to that of tissue regeneration in lower and higher animals, would deserve much attention and should be undertaken because of the potential value if proved successful.

Either tissue-specific normal stem cells or cancer stem cells possess the ability to divide, differentiate or retrodifferentiate [4]. It is of greatest importance to know what homeostatic forces may drive those cells to full differentiate or at least to attain stationery states. It could give the opportunity of neutralising the growing activity of tumours, without the harmful effects derived from the use of cytotoxic drugs and ionising radiations. It might also help to elucidate the serious problems encountered to progress in regenerative medicine.

### Immunotherapy and cancer

The harnessing of patient’s own immune system to fight cancer has attracted the attention of the biomedical word for more than a century. That an immune response to the emergence of cancerous cells exists in animals and men is generally accepted. Great progress has been made on elucidating such an anti-tumour response and the way to activate it with a variety of procedures. Nevertheless, the results obtained in clinical practice were until now rather modest, although some recent successful clinical trials have given hope of promise for the future [56]. The reasons underlying the lack of greater success have been well analysed and explained in a publication from the Society for Immunotherapy of Cancer (SITC) [55] that summarises the discussions and recommendations gained at an “Immunotherapy Submit” with representatives from immunotherapy organisations of different countries. Several hurdles were identified that impede the successful translation of immunotherapy into clinical practice. Among them are as follows: (a) the limitations encountered by the use of animals as preclinical models; (b) the complexity of cancer due to cell heterogeneity and its translation into different clones with different target characteristics; (c) the variability of the anti-tumour response according the status of the patients (age, previous treatments, tumour progression, etc.); (d) the lack of definitive biomarkers of immune response permitting discrimination of what, in a case of tumour regression, is due to immunotherapy and what to previous or simultaneous unrelated treatments (chemotherapy, radiotherapy); and (e) the risk of over-activation of the response to immunotherapy, causing auto-immune harmful reactions.

The use of immunotherapy as a potential weapon against malignancies can be criticised because this kind of treatment belongs to the group of cell-killing therapies. Nevertheless, the fine targeting of cancerous cells via stimulation of the immune system to produce responses against cancer cells should, in contrast to chemotherapy or radiotherapy, exclude from the cytotoxic effects those normal mature or stem elements that are unrelated to cancer. On the other hand, considering our present knowledge of cancer biology and the current status of cancer care as well as the recent popularity of personalised treatments, immune-based cancer therapies deserve greater attention as a possible new and helpful frontier in tumour treatment. Last but not least, the strong relationship between cancer development and the immune response calls for cooperative studies of immunity and cancer biology. It is well known that life is a matter of DNA but survival depends on a well-functioning immune system. Its failure, particularly in cancer patients, triggers rapid deterioration. In fact, progress in cancer knowledge and treatment is probably indebted to progress in cancer immunity. This evidence should be sufficient to justify and stimulate active research work in this area.

## Concluding remarks

Significant progress was made over the last 60 years in the knowledge of cancer biology and treatment of the disease. However, over the same period of cancer research and clinical practice the ultimate “secret” leading to “cure” of malignant tumours has not been found. This is not a single case in modern medicine in the last 50 years.

Thanks to the advances in genomics we know, and it is very important, that sets of spontaneous or induced mutations affecting key genes are probably pivotal in cancer initiation [2]. Once again, great hopes of future advances in cancer management have been associated with this recent discovery. Unfortunately, one might anticipate that if developed alone this new approach would not clear up, at least for the short or middle term, the question of when, why and how one or several normal cells become a malignant neoplasm. Different and still ignored causes or mechanisms of tumour emergence and progression will, probably, be found in the future. Also, the finding of advanced and specific markers for cancer screening might be expected.

Although cell-killing therapies have become paradigmatic in fighting cancer progression, it seems imperative to open new ways of research and to activate underestimated ones. Also, imperative is to continue persuading human populations and governmental agencies that the best line to fight cancer remains avoidance of all external and internal agents contributing to tumour incidence. Among them, long-term and repeated cell injuries are probably the primary accident in the chain of events leading to oncogenesis. All other recourses, although indispensable today, are long, distressing, costly and uncertain.

## Footnotes

1 More recently, the term “reprogramming” has been used to describe the induction, from adult or adult stem cells, of pluripotent stem cells (iPSCs) with high capacity to differentiate in vitro and in vivo [25]. Nevertheless, reprogramming means to program again. Perhaps the term “retroprogramming” could be used with the same meaning as “retrodifferentiation”.
